# In-Series Production of Integrated Bi-Doped Laser-Induced Graphene Sensors for Simultaneous Detection of Zn(II), Cd(II), and Pb(II)

**DOI:** 10.1021/acsmeasuresciau.6c00132

**Published:** 2026-07-13

**Authors:** Pamela Rivera Rivera, Ida Valeria Di Cristoforo, Annalisa Scroccarello, Davide Paolini, Flavio Della Pelle, Rasa Pauliukaite, Dario Compagnone

**Affiliations:** † Department of Bioscience and Technologies for Food, Agriculture and Environment, 19031 University of Teramo, Via R. Balzarini, 1, Teramo TE 64100, Italy; ‡ Department of Nanoengineering, 226274 Center for Physical Sciences and Technology (FTMC), Savanoriu Ave. 231, Vilnius LT-02300, Lithuania; § University School for Advanced Studies IUSS Pavia, Piazza della Vittoria, 15, Pavia 27100, Italy

**Keywords:** nanofilm, zinc analysis, heavy metal detection, sweat analysis, electrochemical device, laser-induced graphene, Bi doping

## Abstract

Laser-induced graphene (LIG) is a breakthrough material for electrochemical sensing, and its sensing ability can be tuned through targeted modifications. Herein, a xurography/laser approach is proposed to fabricate in-series, self-contained LIG sensors incorporating bismuth (Bi-LIG), conceived for the simultaneous detection of Zn­(II), Cd­(II), and Pb­(II). The Bi-LIG sensors were assembled using poly­(vinyl alcohol)-assisted Bi doping and xurography, without the need for polyimide chemical pretreatment. The manufacturing process relies on forming a PVA layer incorporating Bi^3+^ on a polyimide foil via stencil printing, followed by laser-induced formation of the Bi-doped LIG sensing layer, the counter electrode, and contact tracks; the sensors were completed by stencil printing to form reference electrodes and by thermal lamination to insulate. The formation of the Bi-LIG sensing layer was carefully studied, examining the effects of laser power and Bi content. After determining the optimal laser power to form Bi-LIG, Bi precursor concentration revealed a nonmonotonic effect on electrochemical performance, with electrochemical impedance spectroscopy and Raman analysis showing an optimal concentration of 20 mM, characterized by lower charge-transfer resistance and reduced Raman-active disorder. Morphochemical analyses (SEM, EDX, XPS) confirmed the presence of highly dispersed, predominantly oxidized Bi species, with no visible nanoparticle formation. Bi-LIG sensors enabled simultaneous, reproducible detection of Zn­(II), Cd­(II), and Pb­(II) via square-wave anodic stripping voltammetry (RSD ≤ 10%; n = 3), yielding submicromolar LODs (≤0.1 μM) and well-resolved peaks. These sensors achieved quantitative recoveries of the three metals in artificial sweat (Rec 95–112%; RSD ≤ 12%; n = 3).

## Graphical abstract

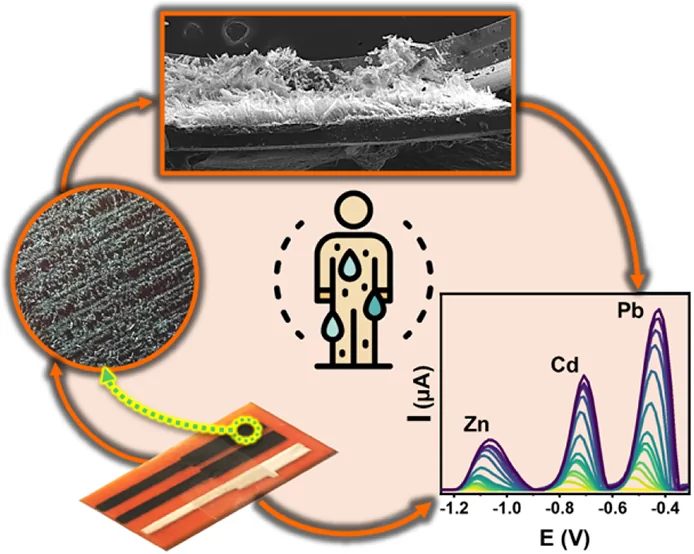

## 1 Introduction

Laser-induced graphene (LIG) has become a versatile platform for the rapid, maskless, and scalable fabrication of conductive carbon structures directly on flexible substrates.^1–4^ LIG produced by CO_2_ laser scribing exhibits a unique combination of high porosity, turbostratic graphene domains, and interconnected conductive pathways, enabling applications in flexible electronics, catalysis, electrochemical sensing, and biosensing.^2,5–11^ Despite its high surface area and good electrical conductivity, LIG has limited catalytic specificity due to its low density and poorly defined electroactive surface sites.^12^ This restricts selective interactions between analytes and the electrode, thereby diminishing electrochemical sensing capabilities. Therefore, surface and composition engineering are necessary to introduce well-defined active sites that promote selective interfacial reactions while preserving the efficient electron-transport properties of the LIG framework.

On the other hand, heteroatom doping is a widely used strategy to modify the electronic structure, catalytic activity, and interfacial properties of graphene-derived materials.^12^ Conventional methods, such as drop-casting nanoparticles, electrodeposition, hydrothermal treatment, and plasma functionalization, typically introduce dopants after the material has been synthesized.^13^ These postprocessing techniques often encounter issues such as weak dopant adhesion, spatial inhomogeneity, and limited reproducibility, which can significantly affect the electron-transfer ability of the sensing surface. In contrast, in situ doping and functionalization during CO_2_ laser graphene formation enable dopants to be chemically incorporated into the carbon matrix as the polymer undergoes photothermal decomposition and graphitization.^14^ In this context, Salama Research Group introduced an approach in which the metal precursor is incorporated into a PVA membrane, which is then adhered to a polyimide substrate. This approach was used to produce copper nanoparticles via laserization processes with nanozymatic activity on LIG, which were used to determine NO_2_ in artificial human serum.^15^ A similar approach was also proposed to produce bimetallic (iron–cobalt) systems on LIG for the same analytical purposes.^16^ In both works, the PVA-metal polymer was spread onto the polyimide film by blade coating.

Heavy-metal detection establishes a stringent electroanalytical standard because reliable stripping responses require clearly defined active sites, rapid electron-transfer kinetics, and stable dopant–carbon interactions at trace levels. Among others, bismuth is a particularly attractive dopant for electrochemical applications. It forms fused alloys with Zn­(II), Cd­(II), and Pb­(II), enhances preconcentration efficiency in anodic stripping voltammetry, and serves as a nontoxic alternative to mercury films.^17,18^ Incorporating Bi into graphenic materials, although potentially promising for creating a high electroactive surface capable of metal accumulation and detection, remains challenging. This task is especially difficult for LIG because laser processing, due to its high-energy irradiation, produces local high temperatures that can cause Bi evaporation and/or displacement.^19^

To the best of our knowledge, only a few studies have reported Bi incorporation into LIG for stripping-voltammetric heavy-metal sensing. Zhao et al.^20^ used a PVP/Bi precursor ink sprayed onto PI, followed by laser conversion to form BiNP@LIG and a subsequent Nafion coating for Cd­(II) and Pb­(II) detection. Yuan et al.^21^ proposed an ion-exchange plating route in which PI was first chemically modified with concentrated KOH, then exposed to Bi­(III), and finally subjected to laser direct writing to obtain N@Bi-LIG microelectrochemical sensors. More recently, Srirabai and Puthongkha^22^ developed a two-step CO_2_ laser strategy in which LIG was first patterned on PI, followed by drop-casting Bi­(III)/NaOH and a second laser-irradiation step to form Bi nanoplatelet-modified LIG. These reports show that Bi incorporation into LIG requires efficient retention of Bi species during laser processing, achieved through polymer-assisted immobilization, chemical ion exchange, or post-LIG Bi modification.

In this work, a stencil-printable PVA/Bi precursor layer is directly applied to PI, enabling simultaneous Bi incorporation and LIG formation without chemical pretreatment of PI, ion-exchange plating, or post-LIG Bi drop-casting. The proposed approach also enables in-series fabrication of complete integrated Bi-LIG sensors and extends the analytical scope to the simultaneous detection of Zn­(II), Cd­(II), and Pb­(II). For the first time, the effect of Bi on LIG’s graphitization, defect density, surface chemistry, and electron-transfer properties was systematically studied to find the best balance between preserving LIG’s electrochemical features and Bi doping. The strategy relies on stencil printing a PVA precursor film onto polyimide, followed by conversion to Bi-doped LIG (Bi-LIG) via CO_2_ laser scribing. Bi doping was systematically studied by varying both the Bi precursor concentration and the laser power, revealing how Bi incorporation modulates LIG microstructure, graphitic ordering, defect density, and surface chemistry, thereby affecting electron-transfer features and sensing performance. Ultimately, the complete Bi-LIG sensors were tested to simultaneously detect Zn­(II), Cd­(II), and Pb­(II) in model solutions and artificial sweat, demonstrating their usability in real-world applications.

## 2 Materials and Methods

### 2.1 Materials, Chemicals, and Samples

Polyimide (PI) film, 0.125 mm thick, was purchased from Goodfellow (Cambridge, UK); stencil foils were obtained from TINYYO (Parson, Wisbech, UK); and 0.125 mm-thick thermo-adhesive laminating sheets were acquired from Fellowes (polyethylene terephthalate-ethylene vinyl acetate; Itasca, Illinois, USA). Additionally, Ag/AgCl ink and poly­(vinyl alcohol) (PVA, Mw 13,000–23,000, 87–89% hydrolyzed) were sourced from Sigma-Aldrich (St. Louis, MO, USA).

Zinc nitrate hexahydrate [Zn­(NO_3_)_2_·6H_2_O, ≥ 96% purity] was obtained from Carl Roth (Karlsruhe, Germany). Sodium acetate, nitric acid (≥69 wt %), cadmium nitrate tetrahydrate [Cd­(NO_3_)_2_·4H_2_O], lead­(II) nitrate [Pb­(NO_3_)_2_], bismuth­(III) nitrate pentahydrate [Bi­(NO_3_)_3_·5H_2_O], uric acid (UA), ascorbic acid (AA), and glacial acetic acid (≥99.7%) were purchased from Sigma-Aldrich (St. Louis, MO, USA). Artificial sweat (AS) was prepared in accordance with EN 1811:2012 using NaCl (0.5% w/v), KCl (0.1% w/v), urea (0.1% w/v), and lactic acid (LA) (0.1% w/v) in deionized water (pH 4.5–5.0). All reagents were of analytical grade and used as received. Solutions were prepared with deionized water (18.2 MΩ·cm, Milli-Q system), unless otherwise specified.

### 2.2 Manufacturing and Characterization Apparatus

The Silhouette Cameo 4 craft cutter (Silhouette America, Lindon, USA), operated by dedicated software, was used to create stencil masks. A Pavo 8038 718 Unbekannt device was employed, and a thermal roll machine was used to insulate the sensors’ contacts via thermal lamination. The Rayjet50 laser plotter from Trotec (Wels, Austria), equipped with a 10.6 μm, 30 W CO_2_ laser and a 0.04 mm laser spot, was used to produce LIG. Adobe Illustrator 2020 was used to design the LIG patterns.

Electrochemical experiments were conducted using a portable Potentiostat/Galvanostat/Impedance Analyzer PalmSens 4 (Palm Instruments BV, Houten, Netherlands) controlled by PS Trace 5.9 software.

Morphological and elemental analyses were performed using scanning electron microscopy (SEM) with a GeminiSEM 500 (Zeiss Co., Oberkochen, Germany) equipped with an Oxford Aztec Live Microanalysis system and an Ultim Max 100 detector (EDS, OXFORD, Buckinghamshire, UK) for energy-dispersive X-ray spectroscopy (EDX).

Raman measurements were performed using a Raman spectrometer/microscope in Via (Renishaw, UK) equipped with a thermoelectrically cooled CCD detector (−70 °C). Spectra were excited at 532 nm and dispersed using an 1800-groove/mm grating. Raman spectra were collected using a 50×/0.75 NA objective lens. Raw data were processed with Origin 2021 Pro software. To ensure consistent comparisons across bismuth concentrations, all spectra were normalized to the G-band intensity. Structural metrics were obtained by spectral deconvolution with a Lorentzian function to resolve the main vibrational modes: the D band (∼1350 cm^–1^), the G band (∼1580 cm^–1^), and the D’ band (∼1620 cm^–1^). The D’ band was deconvoluted separately to distinguish lattice stretching (G) from defect-activated intravalley scattering (D’), ensuring precise measurement of the graphitic signal. The degree of structural disorder and doping-induced lattice modifications was assessed using integrated area ratios (A_D_/A_G_) rather than peak intensities, providing an accurate evaluation of the overall vibrational density of states in the functionalized graphene.

X-ray photoelectron spectroscopy (XPS) measurements were performed on a Kratos Axis Supra spectrometer (Kratos Analytical, Kyoto, Japan) equipped with a monochromatic Al Kα radiation source (1486.69 eV). The C 1s peak at 284.6 eV was used for calibration, and data were collected at a pass energy of 20 eV. Spectra were processed and deconvoluted using CASAXPS software. After subtracting a Shirley-type background, peaks were fitted with symmetric Gaussian–Lorentzian functions.

### 2.3 Bi-LIG Sensors Manufacturing

The main steps in manufacturing Bi-LIG sensors are illustrated in Scheme 1.

**Scheme 1:**
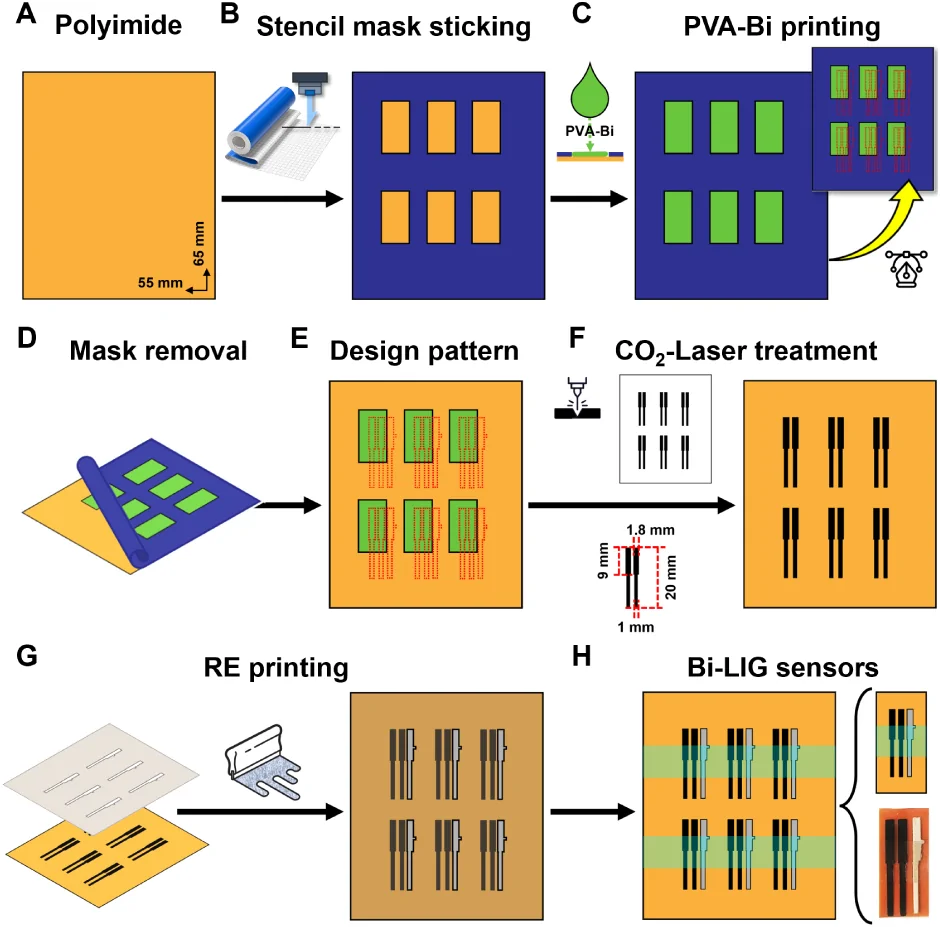
Scheme of the Bi-LIG in Series Manufacturing; (**A**) Polyimide Foil (PI), (**B**) Stencil Mask Stuck onto the PI, (**C**) PVA-Bi Printing, (**D**) Stencil Mask Removal, (**E**) Laser Profile Printing Rendering, Including WE and CE Electrodes, (**F**) CO_2_-Mediated Bi-LIG Formation, (**G**) Pseudo-Ag/AgCl RE Electrode Printing Using the Dedicated PVA Mask, (**H**) Bi-LIG Sensors after Contact Insulation with PET-EVA, with a Picture of the Complete Ready-to-Use Sensor

Before sensor fabrication, a PVA-Bi solution was prepared as a Bi precursor source. 1% m/v PVA was dissolved in deionized water, heated to 70 °C, and stirred until fully dissolved. Then, 2.0 mL of bismuth nitrate pentahydrate (0.1 M), previously dissolved in 1.5 M nitric acid, was added to the PVA solution to reach a final concentration of 20 mM. The mixture was stirred continuously for 30 min to ensure complete dissolution.

(**A**) A PI rectangle was cut to approximately 55 × 56 mm; the PI acts as the LIG precursor and forms the sensor’s base. (**B**) A dedicated stencil mask was designed and created using a cutter plotter, then attached to the PI film. The adhesive mask was designed to facilitate PVA-Bi film printing on the PI within the area that will be converted into working electrodes (WE) and a counter electrode (CE). (**C**) Next, the PVA-Bi dispersion was gently spread onto the PI through the stencil to define the deposition area; 60 μL cm^–2^ was used. The PVA-Bi ink-coated film was then placed on a hot plate and dried at 60 °C for 1 h. (**D**) After drying, the stencil was carefully removed. (**E-F**) Then, the PI film with the dried PVA-Bi layer was laser-engraved. The laser was employed to obtain parallel WE and CE at their point of contact, and to provide a conductive contact for the reference electrode (RE). To this end, the laser engraving mode was employed with a laser power of 24 W, a speed of 1.5 m s^–1^, a z-distance of 6.9 cm, and a resolution of 1000 DPI. In **Panel F**, the geometries and dimensions of the LIG conductive traces are detailed. (**G**) The electrochemical cell was completed by gently brushing the Ag/AgCl ink onto the reference electrode, and the sensors underwent a curing step (60 °C, 10 min). (**H**) Eventually, the electrode contacts were separated from the “sensing part” using dedicated PET-EVA laminating sheets and a thermal laminator. The complete Bi-LIG sensors have the same dimensions as commercial screen-printed electrodes, enabling compatibility with common potentiostat contact connectors, and the insulation was designed to leave out a geometric working electrode surface area of approximately 7.07 mm^2^.

### 2.4 Electrochemical Measurements

CV and EIS measurements were conducted using Bi-LIG as the working electrode, with a Pt wire and Ag/AgCl in 3 M KCl serving as the auxiliary and reference electrodes, respectively. Both measurements were performed in a 5.0 mM [Fe­(CN)_6_]^3 –^ /^4 –^ solution containing 0.1 M KCl. CV was carried out after a 5 s equilibration by sweeping the potential from −0.3 to 0.6 V at a scan rate of 10 mV s^–1^. EIS measurements were taken at the open-circuit potential (OCP) after a 30 s equilibration, applying a 5 mV AC perturbation across the frequency range from 100 kHz to 0.1 Hz (61 points, 10 per decade). The EIS data were analyzed by fitting the experimental Nyquist plots to an equivalent electrical circuit using PStrace software. The circuit included a solution resistance (R_s_) in series with a parallel combination of a constant phase element (CPE), representing the nonideal double-layer capacitance of the porous LIG surface, and a charge-transfer resistance (R_ct_). A Warburg impedance (Z_w_) was added in series with R_ct_ to account for the diffusion-limited processes of the employed redox probe.

The Bi-LIG complete sensor (three-electrode integrated cell) was used to detect heavy metals by square-wave anodic stripping voltammetry (SWASV) in a 0.1 M acetate buffer (pH 4.5) after deaeration with nitrogen gas. The following parameters were set: deposition potential −1.40 V, deposition time 180 s, conditioning potential −0.40 V, conditioning time 6 s, step potential 15 mV, amplitude 40 mV, and frequency 15 Hz. The sensors were used as fabricated, with no pretreatment. All measurements were performed in triplicate.

## 3 Results and Discussion

The idea behind this work was to propose an easy and effective method for producing Bi-LIG sensors for the simultaneous detection of Zn­(II), Cd­(II), and Pb­(II). Therefore, a PVA-film-assisted stencil printing *in situ* strategy is proposed to incorporate Bi into LIG during CO_2_ laser PI conversion, and an accessible xurographic integrated sensor formation approach was undertaken, with particular focus on the effect of Bi integration on sensing capabilities.

### 3.1 Bi-Doped Laser-Induced Graphene Synthesis and Sensing Capabilities

The CO_2_ laser’s ability to convert PI into conductive LIG and simultaneously doped the resulting nanostructured conductive pattern with Bi was initially investigated. Accordingly, the amount of Bi­(III) was examined while the laser parameters were kept constant, consistent with previous studies.^7^

Figure 1A resumes the Bi-LIG formation from the PI precursor enriched with the Bi^3+^ PVA layer; this process is fully reported in Scheme 1 and detailed in Section 2.3. The resulting LIG-only and Bi-LIG were prepared with different amounts of Bi­(III) precursor (from 2 to 40 mM) and analyzed. In particular, a multiscale analysis was conducted to correlate lattice-level Raman signatures, interfacial charge-transfer kinetics, and analytical sensing capabilities. This combined assessment shows a strong nonlinear dependence on Bi loading, indicating that graphenic film performance is affected by interfacial electronic organization rather than by significant bulk structural changes in the carbon framework.

**Figure 1:**
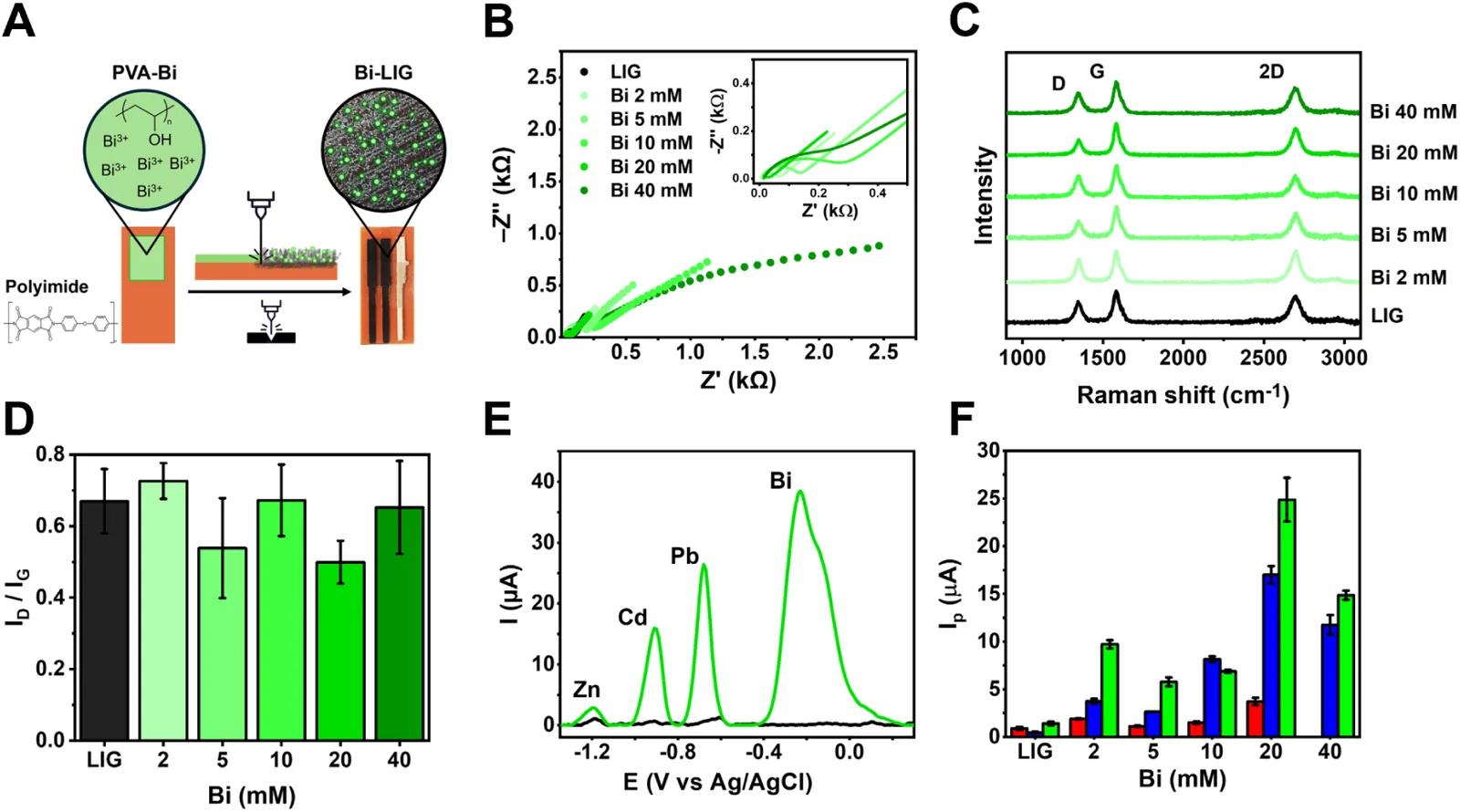
(**A**) Graphical sketch and picture of laser-induced Bi-LIG. (**B**) Nyquist plots obtained for Bi-LIG sensors fabricated with different amounts of Bi precursor; the inset shows a zoom of the EIS spectra fits. (**C**) Raman spectra of Bi-LIG sensors fabricated using different amounts of Bi precursor and the respective (**D**) D and G peak intensity ratios. (**E**) SWAVS peak profile of 10 μM Zn, 10 μM Cd, and 10 μM Pb 0.1 M in acetate buffer (pH 4.5) obtained at the LIG (black line) and Bi-LIG (green line) sensors prepared using 20 mM Bi. (**F**) SWAVS-derived peak intensities of 10 μM Zn (red bars), 5 μM Cd (blue bars), and 5 μM Pb (green bars) obtained with Bi-LIG sensors manufactured with increasing concentrations of Bi.

Figure S1A and Figure 1B show the CVs and the Nyquist plots derived from EIS spectroscopy, respectively, obtained using [Fe­(CN)_6_]^3 –^ /^4 –^ as an electrochemical redox probe. In the CV, a decrease in the faradaic peak intensities is observed with increasing amounts of incorporated Bi; meanwhile, an interesting behavior occurs with the peak-to-peak separation (ΔE), which decreases up to Bi 10/20 mM (120 ± 4 mV) compared to the initial LIG-only sample (149 ± 2 mV), then increases again at Bi 40 mM (157 ± 12 mV). This behavior was more clearly evident in the zoomed Nyquist plot in Figure 1B, where it is apparent that the R_ct_ does not scale monotonically with Bi concentration.

In detail, LIG-only (0 mM Bi) exhibits a baseline R_ct_ of approximately 27 Ω. Upon initial Bi incorporation (2–10 mM), R_ct_ increases progressively, reaching a maximum of ∼266 Ω at 10 mM. This regime is consistent with the presence of electronically isolated Bi domains that may increase interfacial heterogeneity and partially disrupt efficient electron-transfer pathways.^23–25^ A notable decrease in R_ct_ is observed at 20 mM Bi, with the resistance reaching a minimum of ∼17 Ω. This pattern aligns with the formation of more electronically connected Bi domains that can improve interfacial charge-transfer pathways across the LIG surface. At this concentration, the surface density and electronic interaction of Bi species may be enough to reduce interfacial resistance while maintaining the intrinsic conductive framework of the LIG. Higher loading (40 mM Bi) leads to a significant increase in R_ct_ (2173 Ω). This behavior is consistent with an excess of dopant that may enhance interfacial heterogeneity, promote dopant aggregation, and partially block the hierarchical porosity of LIG, potentially leading to mass-transport limitations and hindering the charge transfer.^20^

Figure 1C and D shows the Raman spectra and the corresponding extrapolated I_D_/I_G_ ratio data, respectively. The Raman signature aligns with the interfacial interpretation when analyzed within established frameworks for doped graphenic materials. The I_D_/I_G_ ratio shows a nonmonotonic dependence on Bi concentration, with a minimum at 20 mM (0.51 ± 0.03), along with decreased D′ contributions and narrower G- and 2D-line widths.

In doped carbon systems, Raman disorder metrics reflect a combination of defect-related scattering and dopant-induced electronic effects, rather than serving as a direct measure of only graphitization. Variations in carrier concentration and electronic coherence can significantly alter Raman intensity and line width without requiring extensive lattice reconstruction.^26–28^ Similar nonmonotonic relationships between Raman disorder parameters (I_D_/I_G_, band broadening, and D′ features) and dopant loading have been observed in chemically modified carbon materials, where enhanced electronic coherence and aromatic connectivity—rather than increased structural order—govern charge-transport behavior.^29,30^ Accordingly, the Raman “minimum” observed at 20 mM Bi can be understood as a reduction in Raman-active disorder and improved electronic coupling at the carbon–Bi interface, rather than as direct evidence of extensive restructuring of the bulk carbon lattice.

The sensing performance of Bi-LIG fabricated with different amounts of Bi­(III) precursor was evaluated using SWAVS at fixed concentration of 10 μM of each cation: Zn, Cd, and Pb (measurement parameters detailed in Section 2.4). As an example, Figure 1E shows the voltammogram obtained with the bare LIG and Bi-LIG sensors formed using 20 mM of bismuth precursor; the Bi-LIG sensors display three well-resolved peaks, attributed to Zn (∼−1.2 V), Cd (∼−0.9 V), and Pb (∼−0.7 V); at a more positive potential (∼−0.23 V), the Bi peak is visible. The importance of Bi doping is clear from the LIG-only voltammetric response (black line), which exhibits a nearly flat baseline with no detectable metal signals, demonstrating that Bi doping is essential for achieving sensitivity and peak resolution for the simultaneous detection of these analytes.

Figure 1F shows the peak currents for the target analytes (Zn, Cd, and Pb) measured using Bi-LIG sensors fabricated from PVA Bi films containing different Bi concentrations. All Bi-LIG sensors yielded reproducible signals (RSD ≤ 13%, n = 3), confirming the reliability of the sensor fabrication process. The bar chart shows that 20 mM Bi resulted in higher peak currents across all heavy-metal analytes. Figure S1B shows the Bi peak currents obtained with Bi-LIG sensors; interestingly, the sensor containing 20 mM Bi exhibited a higher peak current for bismuth, indicating a larger charge or more active Bi species. Therefore, the enhanced sensing ability at this Bi concentration (20 mM) likely arises from improved interfacial electron transfer and the formation of bismuth alloys with the analytes, which boosts the preconcentration process. Notably, a decrease in current is observed at 40 mM Bi, suggesting that higher concentrations may overload the surface, forming a thicker and more resistive layer that hampers mass transport and reduces the active area. This effect is especially clear in the complete loss of the Zn peak at higher Bi concentrations. A similar trend has been reported for other electrode systems in the literature. For example, Guo et al.^31^ explained that this phenomenon is associated with the detrimental effect of excessive bismuth film thickness: overly thick films increase mass-transfer resistance, thereby limiting the diffusion of metal ions through the film and reducing the electrochemical response.

The laser effect on Bi-LIG formation was then examined to evaluate its sensing ability, as optimizing laser parameters is crucial for maximizing Bi retention and “conductivity” in the films.^9,24,32^ Figure S2A shows a bar graphs of Bi peak currents recorded using SWAVS after exposing the polyimide film containing 20 mM Bi precursor to different laser powers. Bi-LIG at 12 and 15 W showed relatively high Bi peak currents, likely due to better surface Bi preservation. A decrease in Bi peak current at 18 W may be due to increased Bi loss and less optimal formation of a graphitic structure. At 24 W, the Bi peak current was highest, indicating an ideal balance between Bi retention and graphene's electrochemical properties, which is important for sensing. Higher laser power resulted in lower Bi signals, likely due to precursor displacement and reduced graphitization efficiency. Figure S2B also shows the peak currents for the target analytes (Zn, Cd, and Pb) measured with Bi-LIG prepared at the studied laser powers, demonstrating that the highest peak currents for all three heavy metals were achieved at 24 W; this laser effect was especially important for Zn sensing.

Based on the collected data, the sensor prepared at 24 W in the presence of a 20 mM Bi precursor provided the optimal balance between high sensing activity and Bi loading while preserving LIG’s electrochemical properties; the complete procedure and laser parameters used for its production are reported in Section 2.3. This sensor was selected for further study and will be referred to as Bi-LIG from now on. For clarity, it is worth noting that the synthesis of Bi-LIG was also attempted in the absence of PVA by depositing and drying the Bi precursor directly onto the polyimide. The results showed unreliability and a limited ability to detect Zn­(II), Cd­(II), and Pb­(II), lower than that obtained using unmodified LIG, demonstrating the active role of PVA in quantitatively and uniformly incorporating Bi into the LIG. However, it is difficult to compare the influence of the polymer matrix with data in the literature. For example, Zhao et al.^20^ used PVP to link Bi­(III), but the laser parameters were adjusted differently for that case. However, the cited study showed that the electrochemical performance of unmodified LIG was significantly better than that of BiNP@LIG prepared using PVP. Therefore, the role of Bi doping in this paper is unclear. In contrast, Bi-LIG outperformed bare LIG in PVA. Nevertheless, the sensitivity to Cd­(II) and Pb­(II) was more than 100 times higher when PVP was used. This difference may not be directly related to the linker polymer itself, but rather to the laser parameters optimized in each study.

### 3.2 Bi-LIG Morphochemical Features

To shed light on the structural and chemical features of the Bi-LIG films, optical microscopy, SEM, EDX, and XPS were conducted. Figure 2A shows the macroscopic features of Bi-LIG, appearing as a dense framework of highly expanded graphene flakes. This morphology is further confirmed by the SEM micrograph of the Bi-LIG cross-section (Figure 2B), from which the LIG expansion and the typical 3D texture are evident. Detailed SEM observations (Figure 2C and D) reveal the formation of a continuous film with a porous carbon morphology typical of laser-induced graphene produced by CO_2_ laser scribing of PI, consistent with rapid photothermal decomposition during laser processing.^33–35^ The graphenic nature of the film, as indicated by the Raman data in Section 3.1, is characterized by a wrinkled surface with graphenic exfoliated flakes out of the plane, organized into a continuous nanonetwork, especially evident in the magnification shown in Figure 2D.

**Figure 2:**
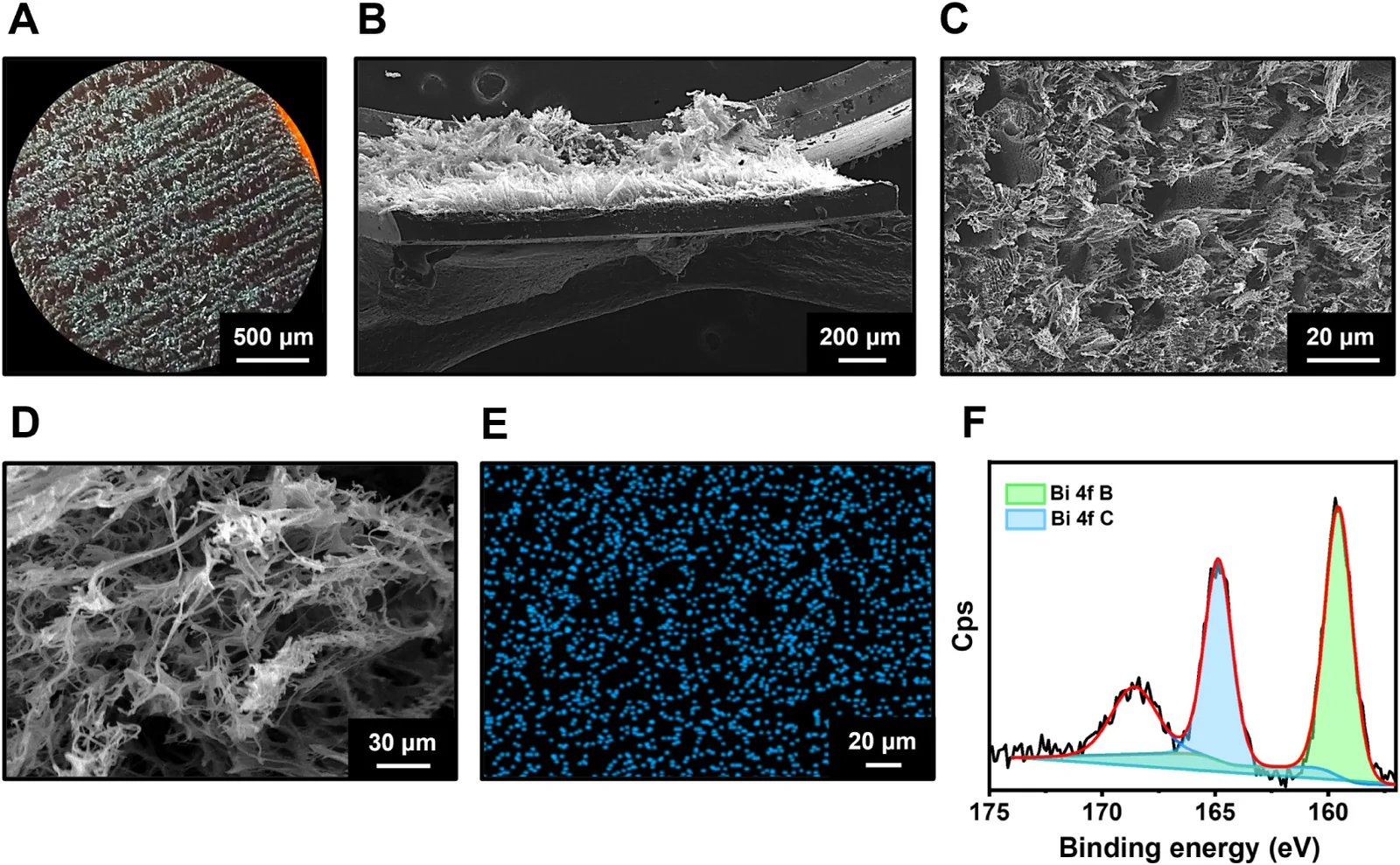
Morphological characterization of the Bi-LIG performed with (**A**) optical microscope and SEM at magnification of (**B**) 50 x, (**C**) 5 kx, and (**D**) 1 kx. (**E**) EDX mapping for Bi of the Bi-LIG. (**F**) XPS high-resolution spectrum of Bi 4f for the Bi-LIG.

Notably, SEM characterization shows an interconnected graphene network without discrete Bi nanoparticles (NPs) or crystalline aggregates. This outcome contrasts with Zhao et al., in which laser conversion of a Bi precursor on PI produced visible Bi NPs.^20^ These findings align with the high-dispersion morphology reported by Yuan et al., who used an ion-exchange plating method to achieve homogeneous bismuth integration onto graphene.^21^ The absence of clustering in Bi-LIG likely results from the stabilizing effect of the PVA matrix; recent literature on NP stabilization suggests that the viscosity and spatial confinement of PVA inhibit metal agglomeration during rapid photothermal conversion.^36^

EDX elemental mapping depicted in Figure 2E confirms the presence and even distribution of Bi across the LIG surface, despite the lack of visible Bi “particles”. The widespread Bi signal suggests that bismuth remains in a highly dispersed, non-nanoparticulate form rather than forming localized clusters; as expected, the Bi particles are in oxide state due to the presence of oxygen elements, complementary to the Bi locations, in the carbonic framework (Figure S3). The chemical state of the Bi was confirmed using XPS; the spectrum in Figure 2F further verified the presence of bismuth on the surface, with the Bi 4f doublet at 160 and 165 eV, which are assigned to the Bi 4f_7/2_ and Bi 4f_5/2_ states of Bi^3+^ ions, corresponding to Bi–O bonds in Bi_2_O_3_ ^22,37–39^ No significant signal from metallic Bi was observed, indicating that bismuth mainly exists in oxidized, surface-accessible chemical forms. However, it is not entirely clear whether bismuth is present in an oxidized form as a partial oxide linked to graphene defects via oxygen bridges or simply incorporated into LIG as independent Bi_2_O_3_. This ambiguity is not unique to the present system: Yuan et al.,^21^ who prepared Bi-doped LIG via ion exchange plating on polyimide, reported essentially identical Bi 4f XPS signatures and similarly found no discrete Bi nanoparticles by TEM, proposing subnanometer incorporation of Bi into the graphene framework.

In this strategy, PVA serves as a polymeric matrix that both immobilizes and disperses the Bi precursor prior to laser treatment. As a water-soluble, hydroxyl-rich polymer, PVA can promote the formation of a homogeneous Bi-containing film on PI, improve precursor retention during drying, and limit Bi aggregation during CO_2_ laser conversion. This interpretation aligns with the known use of PVA as a nonionic stabilizer in nanoparticle preparation, where it adsorbs to particle surfaces, provides steric stabilization, and reduces agglomeration through hydrogen-bonding interactions.^40^ Although this role is related to that of PVP in the BiNP@LIG strategy reported by Zhao et al.,^20^ the two polymers and fabrication routes are not directly comparable. PVP can act not only as a stabilizer but also, in some cases, as a mild reductant that influences nanoparticle formation. Moreover, the properties of metal-doped LIG depend strongly on the overall fabrication methodology, including precursor deposition, laser-processing conditions, post-treatment, and sensor architecture. Therefore, we do not claim that PVA is intrinsically superior to PVP. Rather, our results demonstrate that PVA is well-suited for producing a homogeneous, stencil-printable Bi precursor layer compatible with pretreatment-free, in-series fabrication of integrated Bi-LIG sensors by CO_2_ laser scribing.

### 3.3 Bi-LIG-Based Zn, Cd, and Pb Cations Simultaneous Analysis

Bi-LIG parameters for the detection method were optimized to maximize response and resolution for the simultaneous sensing of Zn­(II), Cd­(II), and Pb­(II). Among these, Zn­(II) has the lowest sensitivity and is the most difficult to detect because its reduction potential (vs Ag/AgCl) lies near the onset of the hydrogen evolution reaction (HER), resulting in a narrow overpotential window and lower accumulation efficiency. Therefore, optimization focused on enhancing or preserving the Zn­(II) response by adjusting SWAVS amplitude (mV), frequency (Hz), and step potential (E step, mV), as these significantly influence peak resolution—an essential factor for simultaneous detection; tests were performed using the Bi-LIG sensor while keeping constant accumulation time and Zn­(II), Cd­(II), and Pb­(II) amounts.

Figure 3A displays the study conducted at scanning amplitudes from 10 to 50 mV, where the peak currents consistently increased with amplitude for all analytes, reaching a plateau at 40 mV. Therefore, 40 mV was selected as the optimal amplitude, balancing signal strength with the need to prevent peak broadening that could reduce voltammetric resolution.^41^ The initial rise in peak current with increasing pulse amplitude in SWV results from the improved driving force for electron transfer under kinetic control. Once the pulse amplitude reaches a level where the reaction is limited by mass transport and reverse current contributions become significant, further increases do not produce higher currents, leading to a response plateau, as observed in this experiment.^42^

**Figure 3:**
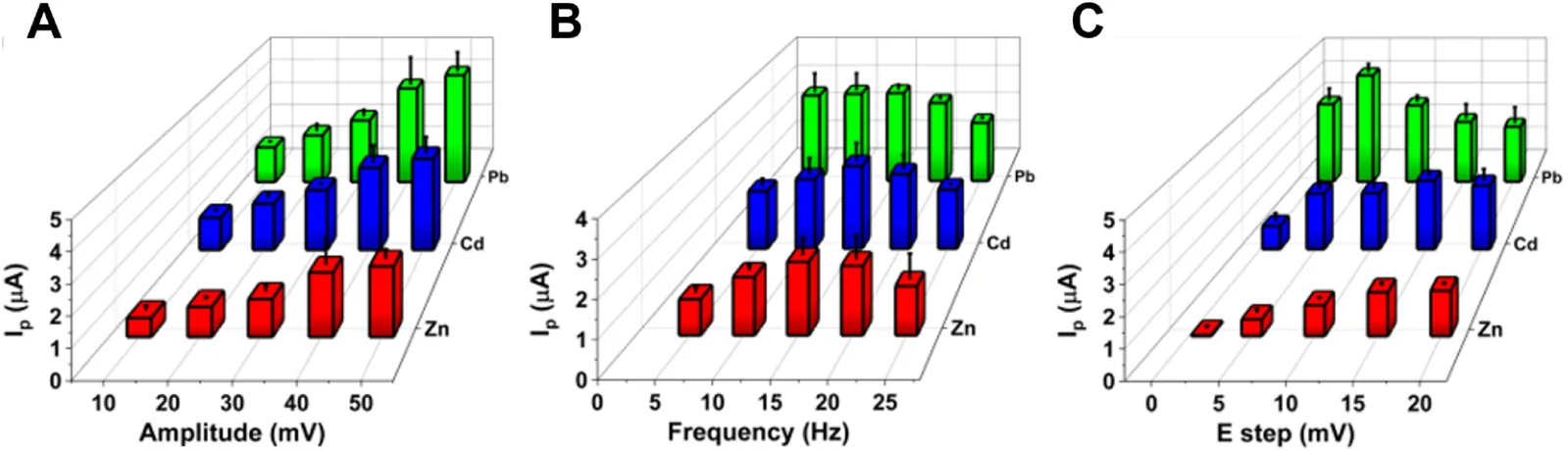
SWAVS parameter optimization for Zn (red), Cd (blue), and Pb (green) simultaneous analysis. Peak currents (I_o_) extrapolate from SWAVS analysis performed by varying (**A**) pulse amplitude (10–50 mV), (**B**) frequency (5–25 Hz), and (**C**) potential step (2–20 mV), analyzing simultaneously 2.0 μM Zn, 1.0 μM Cd, and 1.0 μM Pb in 0.1 M acetate buffer (pH 4.5).

Frequency was tested between 5 and 25 Hz. Figure 3B shows that the peak current increased up to 15 Hz for all analytes and then decreased; thus, 15 Hz was chosen as the optimal frequency. In SWV, frequency is the number of square-wave cycles per second and affects pulse duration and sampling time. The maximum at 15 Hz suggests that the current is sampled after the capacitive current decays, while the faradaic current remains near its maximum, influenced by electron-transfer kinetics and mass transport. Lower currents at lower frequencies result from longer sampling times, during which the faradaic current decays, whereas higher frequencies yield shorter sampling times, leading to a less-developed faradaic current and significant capacitive contributions. These findings align with the quasi-reversible maximum described by Komorsky-Lovrić and Lovrić.^43^

Figure 3C shows how the step potential (ΔE) affects the peak current for Pb­(II), Cd­(II), and Zn­(II). The highest peak current for Pb­(II) occurs at 5 mV, whereas Cd­(II) rises quickly to that point and then increases only slightly at higher ΔE. In contrast, Zn­(II) steadily increases in peak current until it levels off at 15 mV. The dependence of peak current on ΔE for all three metals suggests quasi-reversible behavior, since reversible systems usually show little change with ΔE shifts.^44,45^ These patterns reflect differences in deposition efficiency across metals. Pb­(II) deposits efficiently at higher overpotentials, yielding strong peak currents even with small ΔE. Zn­(II), which deposits near the HER potential, shows poor deposition efficiency and requires larger ΔE to produce measurable currents. Cd­(II) shows intermediate behavior, consistent with its reduction potential lying between those of Zn­(II) and Pb­(II).

### 3.4 Bi-LIG Analytical Features and Application

The analytical performance of the Bi-LIG sensors for simultaneous SWAVS detection of Zn­(II), Cd­(II), and Pb­(II) was evaluated using mixed standard solutions containing all three metal ions. The resulting stripping voltammograms and calibration plots are displayed in Figure 4A and B, and the analytical figures of merit are summarized in Table 1.

**Figure 4:**
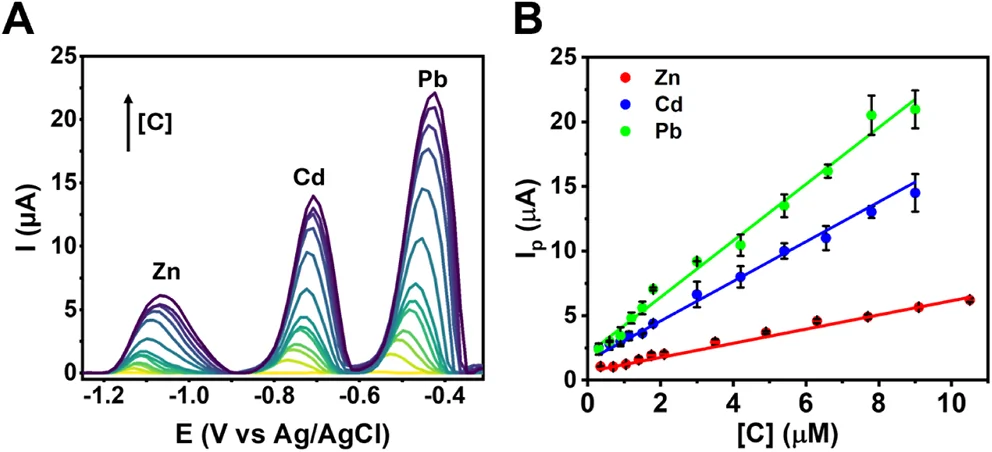
(**A**) SWAVS voltammograms obtained by measuring simultaneously increasing Zn (cyan), Cd (violet), and Pb (maroon) concentrations. (**B**) Calibration curves for Zn­(II) (linear range: 0.35–10.5 μM; y = 0.530 ± 0.017 x + 0.891 ± 0.089; R^2^ = 0.990), Cd­(II) (linear range: 0.30–9.0 μM; y = 1.428 ± 0.029 x + 1.873 ± 0.141; R^2^ = 0.992), and Pb­(II) (linear range: 0.30–9.0 μM; y = 2.189 ± 0.074 x + 2.041 ± 0.339; R^2^ = 0.989).

**Table 1:** Bi-LIG Analytical Figures of Merit for Zn­(II), Cd­(II), and Pb­(II) Simultaneous Detection

Analyte	Linear range	R^2^	Slope (S)	Intercept	σ (Blank)	LoD	LoQ
	(μM)		(μA cm^–2^ μM^–1^)	(μA)	(μA)	(nM)	(nM)
Zn(II)	0.35 – 10.5	0.990	5.9 ± 0.2	0.89 ± 0.09	0.0186	104	314
Cd(II)	0.3 – 9	0.992	15.9 ± 0.3	1.87 ±0.14	0.0072	14	42
Pb(II)	0.3 – 9	0.989	24.3 ± 0.8	2.04 ±0.34	0.0273	37	111

Figure 4A shows well-defined, clearly separated stripping peaks for Zn­(II), Cd­(II), and Pb­(II) at −1.1 ± 0.02 V, −0.74 ± 0.02 V, and −0.47 ± 0.04 V (vs pseudo Ag/AgCl), respectively. As the concentrations of these three metal ions increase, their peak currents rise proportionally, indicating a stable electrochemical response under multimetal conditions in the acetate buffer, thereby enabling simultaneous detection of these metals. The slight anodic shift observed for all the peaks, confirming the formation of intermetallic compounds, is reported in similar systems using SWAVS.

Figure 4B reports the linear calibration plots obtained from SWAVSs: for Zn­(II) over 0.35–10.5 μM (R^2^ = 0.990), Cd­(II) over 0.3–9.0 μM (R^2^ = 0.996), and Pb­(II) over 0.3–9.0 μM (R^2^ = 0.989). Sensitivities followed the order Pb > Cd > Zn, consistent with their stripping behaviors. Despite the simultaneous presence of multiple analytes, there was no significant peak overlap or signal suppression within the tested concentration ranges. Limits of detection (LoD) and quantification (LoQ) were calculated using the 3.3σ/S and 10σ/S criteria, respectively, where S is the slope and σ is the standard deviation of the mean of blank determinations. LoDs of 104 nM for Zn (II), 14 nM for Cd­(II), and 37 nM for Pb­(II) were obtained; LoQs and other figures of merit and parameters are reported in Table 1.

Calibration plots show nonzero intercepts, especially for Cd­(II) and Pb­(II); this behavior is more characteristic of *ex situ* Bi films and is associated with differences in the mechanism of alloy formation relative to *in situ* Bi deposition.^46^ The detection limits are not among the lowest reported for heavy-metal electrodes; they are sufficient to demonstrate reliable, reproducible simultaneous detection under multianalyte conditions and are suitable for disposable, integrated sensors produced through a straightforward in-series approach. The consistency of the measurements and the Bi-LIG manufacturing process was verified by device-to-device reproducibility, evaluated by analyzing the slopes of linear calibration curves for each analyte across three independent calibrations, with an overall RSD ≤ 6.0% (n = 3), indicating system robustness. Overall, the data confirm that the Bi-LIG sensor enables robust simultaneous detection of Zn­(II), Cd­(II), and Pb­(II) in acetate buffer, emphasizing operational simplicity and effective peak discrimination while achieving low detection limits.

The well-resolved stripping peaks observed for Zn­(II), Cd­(II), and Pb­(II) arise from the combined effects of the porous LIG framework, Bi incorporation, and optimized SWAVS parameters.^47^ In SWAVS, sensitivity is inherently enhanced by the preconcentration step, during which metal ions are reduced and accumulated at the working electrode surface, followed by oxidative stripping during the voltammetric scan. The 3D LIG network provides a conductive, high-surface-area carbon framework that facilitates this accumulation and supports efficient charge transfer. The presence of Bi further enhances the stripping response because bismuth-based electrodes interact favorably with heavy metals, forming fused alloys or intermetallic phases during deposition. After accumulation, Zn, Cd, and Pb are stripped at distinct oxidation potentials, yielding separate analytical peaks. Therefore, the peak resolution observed for Bi-LIG results from the combination of Bi-assisted accumulation, distinct stripping potentials of the target metals, and optimized deposition/SWAVS conditions that improve peak definition and minimize signal overlap.^17,18^

Sweat, as a biological fluid, offers a noninvasive means of monitoring biochemical parameters of the human body, including heavy metal exposure. Early research indicated that Zn, Cd, and Pb can be found in sweat at levels similar to those in blood serum, and sometimes even higher than in urine—particularly in individuals with higher body burdens or occupational exposure.^48^ Therefore, to assess how well the Bi-LIG sensors perform under real-world conditions, the detection of Zn­(II), Cd­(II), and Pb­(II) in artificial sweat was evaluated. Thus, before analyzing sweat, the effects of common inorganic ions and organic species typically found in sweat were examined to assess the proposed sensor’s selectivity under simultaneous-detection conditions. The potential interfering compounds were tested at physiological levels^49–51^ analyzing Zn­(II), Cd­(II), and Pb­(II) together with the potential interferents, and the normalized current responses (I/I_0_) obtained were compared to those measured in the absence of interferents.

Figure 5A shows that alkali and alkaline earth metal ions (Na^+^, K^+^, and Mg^2+^) cause only minor changes in the stripping currents of Zn­(II), Cd­(II), and Pb­(II), with signal variations remaining close to one. Similarly, adding LA, uric acid (UA), and ascorbic acid (AA) results in only slight signal deviations, indicating that common electroactive organic compounds found in sweat do not significantly affect the simultaneous detection of the target metal ions or the reproducibility of the sensing (RSD ≤ 12%, n = 3). The slightly larger fluctuations observed for Fe^3+^ and AA are due to their redox activity and possible interactions with the electrode surface; however, these did not cause consistent signal suppression or enhancement.

**Figure 5:**
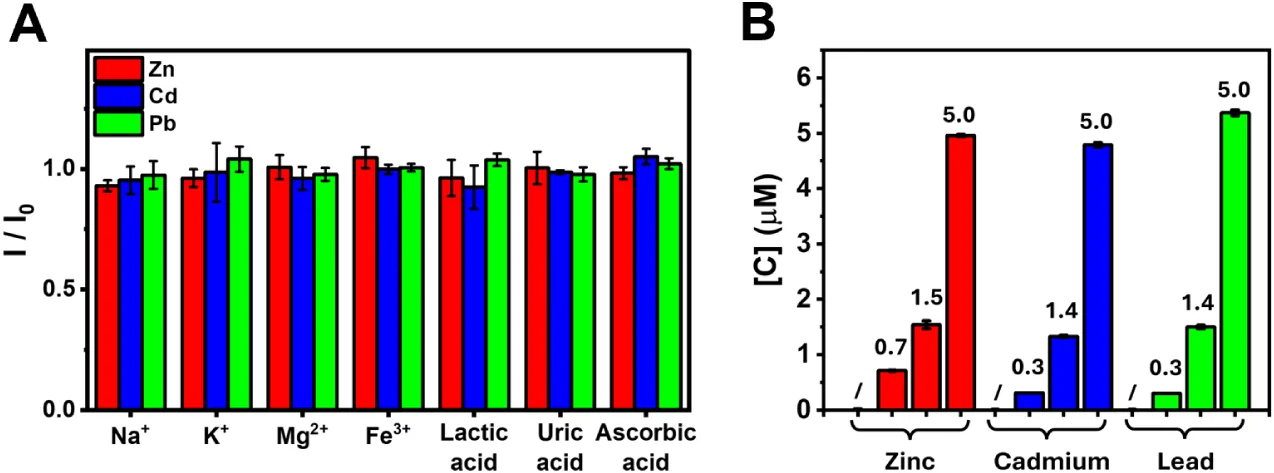
(**A**) Normalized current responses (I/I_0_) derived from SWAVS simultaneous analysis of 1.5 μM Zn, 1.5 μM Cd, and 1.5 μM Pb in 0.1 M acetate buffer (pH 4.5), performed in the presence of potential interferents (30 μM of Na^+^, K^+^, Mg^2+^ and Fe^3+^, 100 μM LA, 10 μM UA, and 10 μM AA; values intended in the measured sample). Error bars indicate SD (RSD ≤ 12%; *n* = 3). (**B**) Zn, Cd, and Pb quantification values extrapolated from artificial sweat analysis spiked at different concentration levels (written on top of the columns).

Eventually, the Bi-LIG working ability in a complex matrix was evaluated through recovery experiments conducted in 10% (v/v) artificial sweat diluted in acetate buffer. Three known combinations of Zn­(II), Cd­(II), and Pb­(II) concentrations were spiked into the artificial sweat, then diluted, and the resulting concentrations were measured using the simultaneous SWAVS detection protocol. The results are shown in Figure 5B and detailed in Table S1. Recoveries for Zn­(II) ranged from 99% to 103%, with RSDs of 3–9%. Cd­(II) recoveries varied from 95% to 112%, with RSDs of 5–12%, while Pb­(II) recoveries were between 107% and 108%, with RSDs of 6–8%. Slightly higher deviations observed at the lowest concentration levels can be attributed to matrix effects and the greater influence of background contributions near the detection limit.

The selectivity of Bi-LIG toward Zn­(II), Cd­(II), and Pb­(II) should be understood as preferential electrochemical accumulation and stripping behavior rather than molecular recognition. Under the selected deposition potential and electrolyte conditions, Zn, Cd, and Pb are efficiently accumulated at the Bi-containing LIG surface and oxidized within distinct potential windows. In contrast, the tested interfering species either do not deposit efficiently under these conditions, are less likely to form comparable Bi–metal phases, or do not produce stripping peaks overlapping with the selected analytical windows. Thus, the good selectivity observed here reflects the combined contribution of Bi-assisted heavy-metal preconcentration, the potential separation of Zn, Cd, and Pb stripping processes, and the optimized SWAVS protocol.

The encouraging data obtained should be viewed as a proof of concept, demonstrating that the proposed sensor maintains detectable analytical performance in a biologically relevant matrix. While this study does not constitute full validation with real samples, it demonstrates the sensor’s potential for the simultaneous detection of heavy metals in biologically relevant media. The next step toward Bi-LIG validation will involve testing with actual contaminated samples and comparing the results with a reference method.

To further contextualize the analytical and manufacturing features of the proposed Bi-LIG sensors, the present approach was compared with previously reported Bi-doped LIG platforms for heavy-metal detection. Table S2 summarizes the key differences in Bi incorporation strategy, pretreatment requirements, processing steps, sensor integration, analytical performance, reproducibility, recovery, and sample application. This comparison shows that, although previously reported Bi-LIG systems achieved excellent Cd­(II) and Pb­(II) detection, the present strategy combines a pretreatment-free, PVA-assisted Bi incorporation route with in-series fabrication of complete, integrated sensors and expands the analytical scope to the simultaneous detection of Zn­(II), Cd­(II), and Pb­(II).

## 4 Conclusions

An easy in situ strategy is proposed to incorporate Bi into LIG during CO_2_ laser conversion of polyimide, thereby creating fully lab-made electrochemical sensors capable of simultaneous detection of metals. The method uses a xurographic approach to print PVA film containing a bismuth precursor, followed by a single-step laser scribing that produces a conductive, highly nanostructured graphenic framework doped with Bi, forming an active sensing surface for Zn­(II), Cd­(II), and Pb­(II) detection. The Bi-LIG sensors were produced in series using a semiautomatic process based on stencil printing and thermal lamination, resulting in sensors that are easy to operate without postsynthesis modification or activation. The best Bi-LIG configuration shows that sensor performance is mainly driven by interfacial electronic organization rather than bulk structural changes in the carbon framework. Morphochemical analysis attributes the Bi-LIG sensing ability to highly dispersed oxidized Bi species, highlighting the importance of precursor chemistry on dopant distribution.

Bi-LIG complete sensors were tested for the simultaneous detection of Zn­(II), Cd­(II), and Pb­(II), achieving effective peak resolution and nanomolar detection limits (LODs ≤ 104 nM). Ultimately, the sensors’ practical application was demonstrated by detecting metals in artificial sweat, showing that they maintained functionality and reproducibility in a simplified biological matrix. Overall, this study proposes a reliable method for tuning metal-doped LIG surfaces and clarifies the structure–property relationships involved in in situ dopant incorporation during laser processing, providing an easy way to produce ready-to-use sensors in series capable of detecting metal cations simultaneously.

## Supplementary Material



## Notes

Figure S1. Bi amount effect of Bi-LIG sensors. Figure S2. Laser power effect on Bi-LIG manufacturing. Figure S3. Bi-LIG EDX mapping. Table S1. Zn­(II), Cd­(II), and Pb­(II) determination in sweat. Table S2. Comparison with Bi-doped LIG sensors for heavy-metal detection (PDF)
