# Supplementary material for: In-Series Production of Integrated Bi-Doped Laser-Induced Graphene Sensors for Simultaneous Detection of Zn(II), Cd(II), and Pb(II)
Source: ACS Meas Sci Au. 2026 Jul 13;6(4):1315–25. doi: 10.1021/acsmeasuresciau.6c00132 (PMC13495726; doi:10.1021/acsmeasuresciau.6c00132)
Supplement: Supplementary file 1 [file tg6c00132_si_001.pdf]

SUPPORTING INFORMATION

In series production of integrated Bi-doped Laser-Induced Graphene sensors for simultaneous detection of Zn(II), Cd(II) and Pb(II)

Pamela Rivera Rivera[a,b,†], Ida Valeria Di Cristoforo[a,c,†], Annalisa Scroccarello[a,†], Davide Paolini[a], Flavio Della Pelle[a,*], Rasa Pauliukaite[b,**], Dario Compagnone[a]

[a] *Department of Bioscience and Technologies for Food, Agriculture and Environment, University of Teramo, Via R. Balzarini, 1, 64100 Teramo TE, Italy*

[b] *Department of Nanoengineering, Center for Physical Sciences and Technology (FTMC),Savanoriu ave. 231, LT-02300 Vilnius*

c University School for Advanced Studies IUSS Pavia, Piazza della Vittoria, 15, 27100 Pavia, Italy

[†] P.R.R, I.V.D.C, and A.S contributed equally to this paper

Corresponding authors:

* fdellapelle@unite.it (F. Della Pelle); ** rasa.pauliukaite@ftmc.lt (R. Pauliukaite).

TABLE OF CONTENTS

**SUPPORTING FIGURES**	**Pg.**
**Figure S1.** Bi amount effect of Bi-LIG sensors	**S3**
**Figure S2.** Laser power effect on Bi-LIG manufacturing	**S4**
**Figure S3.** Bi-LIG EDX mapping	**S5**
**SUPPORTING TABLES**	
**Table S1**. Zn(II), Cd(II), and Pb(II) determination in sweat	**S6**
**Table S2.** Comparison with Bi-doped LIG sensors for heavy-metal detection	**S7**

SUPPORTING FIGURES

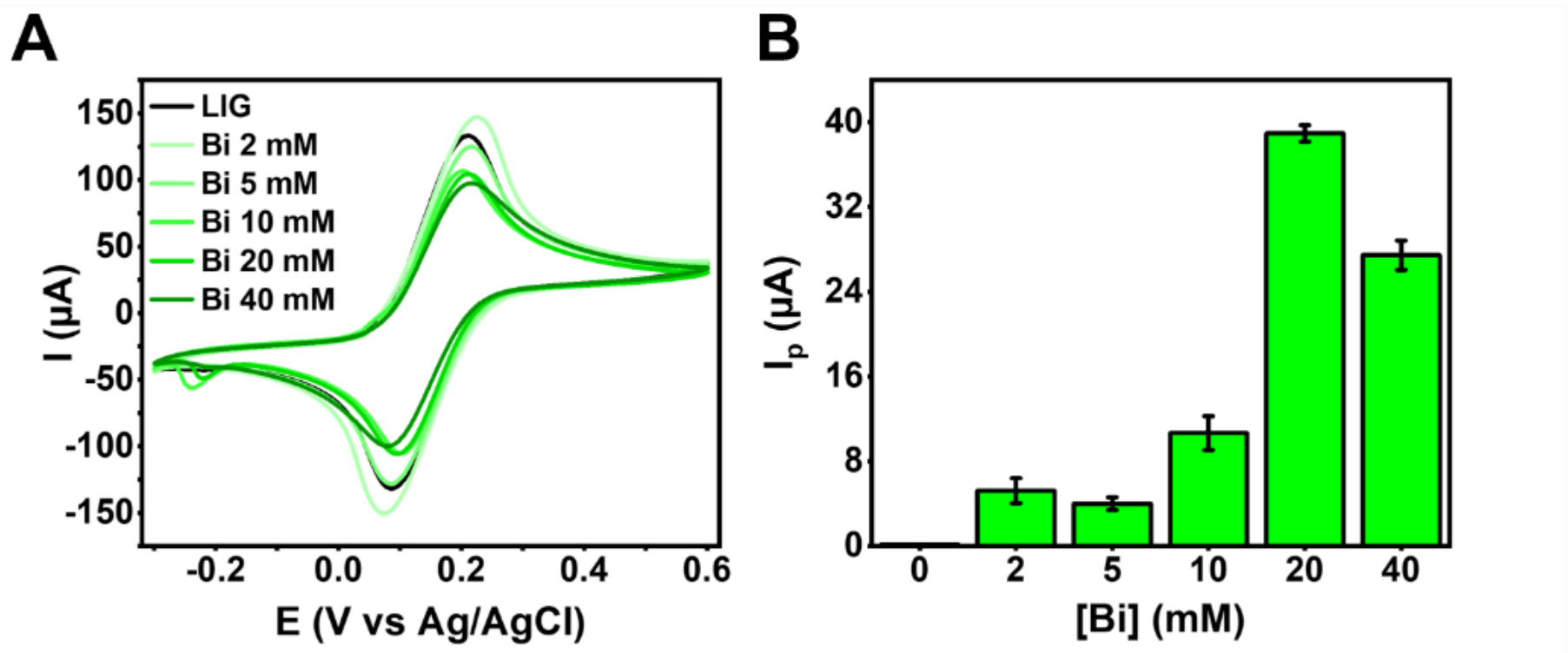

Figure S1. (**A**) Cyclic voltammograms obtained for Bi-LIG electrodes fabricated with different amounts of Bi precursor in 5 mM $[Fe(CN)_6]^{3-/4-}$ prepared in 0.1 M KCl. (**B**) SWAVS-derived peak intensity of Bi-LIG electrodes fabricated with different amounts of Bi precursor in 0.1 M acetate buffer (pH 4.5).

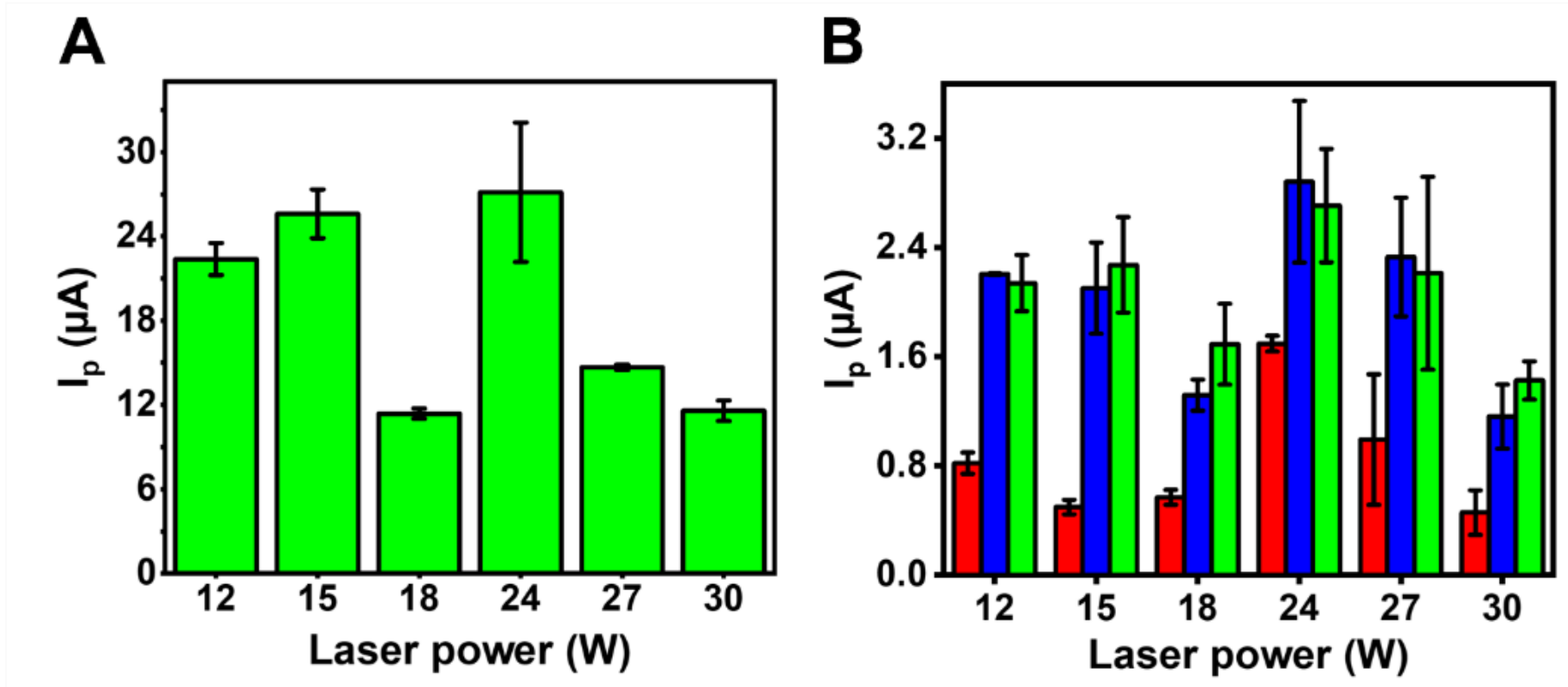

Figure S2. Bi-LIG electrodes fabricated using different laser powers. (**A**) Bi peak currents obtained using SWAVS analysis in 0.1 M acetate buffer (pH 4.5). (**B**) Peak intensity of 1.0 μM Zn, 1.0 μM Cd, and 1.0 μM Pb obtained with Bi-LIG sensors via SWAVS analysis in 0.1 M acetate buffer.

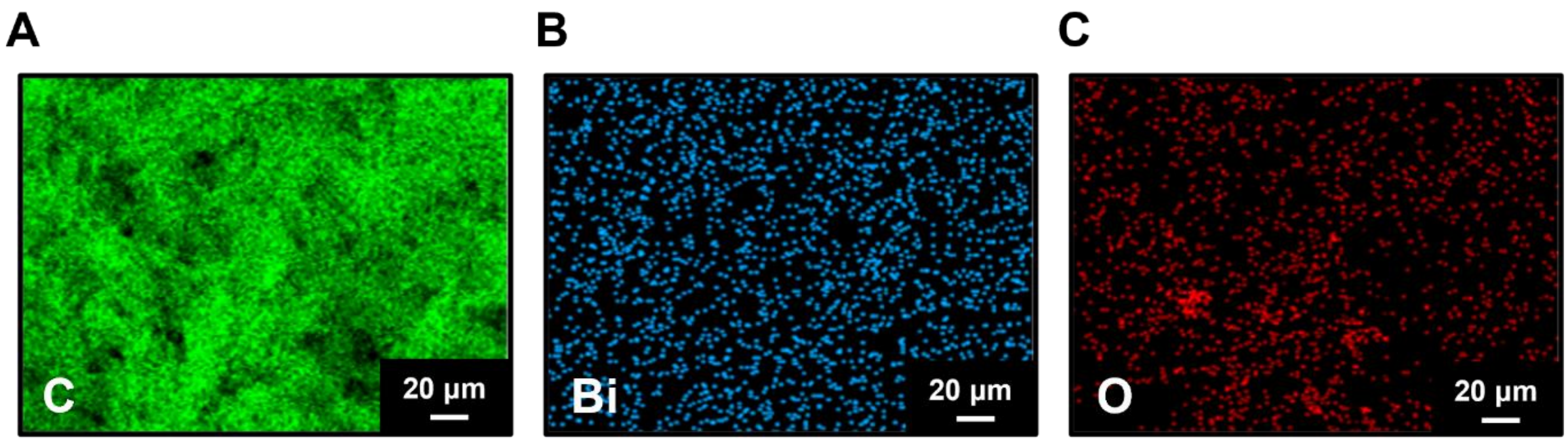

Figure S3. EDX mapping of Bi-LIG, pointing out the (**A**) carbon, (**B**) bismuth, and (**C**) oxygen.

SUPPORTING TABLES

Table S1. Zn(II), Cd(II), and Pb(II) determination in sweat, data expressed as mean value (n=3).

**Analyte**	**Spiked** (μM)	**Found** (μM)	**Recovery** (%)	**RSD** (%)
**Zn(II)**	0	/	/	/
	0.70	0.71 ± 0.06	101	9
	1.50	1.54 ± 0.11	103	8
	5.00	4.96 ± 0.15	99	3
**Cd(II)**	0	/	/	/
	0.28	0.31 ± 0.019	112	5
	1.40	1.33 ± 0.095	95	6
	5.00	4.79 ± 0.70	96	12
**Pb(II)**	0	/	/	/
	0.28	0.30 ± 0.02	108	6
	1.40	1,50 ± 0.15	107	8
	5.00	5.37 ± 0.44	107	7

Table S2. Comparison of the present Bi-LIG sensor with previously reported Bi-doped LIG sensors for heavy-metal detection.

Ref.	Bi-incorpora	Pretreatment/post-treatment	Laser route	Sensor integration	Target analyt	Analytical performan	Reproducibility/recovery	Main distinction
20	PVP-assisted Bi precursor ink sprayed on PI; BiNP@LIG formed by laser irradiation	Nafion coating after laser processing	470 nm laser-induced surface modification	Working electrode coupled with external Ag/AgCl and Pt electrodes	Cd(II), Pb(II)	Linear range 5–50 µg/L (or 44.6–446. nM Cd(II) and 24.2–242 nM Pb(II)); LOD: 0.5 µg/L (4.5 nM) Cd(II), 0.8 µg/L (3.9 nM) Pb(II)	Soil samples; recovery close to ICP-MS values	High Cd/Pb sensitivity; requires Nafion coating; does not include Zn
21	KOH-assisted PI modification followed by Bi ion-exchange plating	4 M KOH pretreatment; $BiCl_3$ ion exchange	UV laser direct writing	Portable micro-electrochemical sensor	Cd(II), Pb(II)	Linear range 1–100 µg/L (or 8.9–892 nM Cd(II) and 4.8–483 nM Pb(II)); LOD: 0.21 µg/L (1.8 nM) Cd(II), 0.410 µg/L (2.0 nM) Pb(II)	RSD 3.1% Cd(II), 3.0% Pb(II); recovery 95.3–106.3%	Excellent Cd/Pb detection; requires alkaline PI pretreatment and ion-exchange step
22	Bi(III)/NaOH dispersion drop-cast onto preformed LIG; second laser step forms Bi nanoplatelets	Post-LIG Bi drop-casting; tape insulation	Double CO_2 laser writing	Single-device three-electrode LIG platform	Cd(II), Pb(II)	Linear range 5–100 ppb (or 0.04–0.89 nM Cd(II) and 0.02–0.48 nM Pb(II)); LOD: 0.25 ppb (2.2 pM) Cd(II), 0.30 ppb (1.2 pM) Pb(II)	RSD 3.50% Cd(II), 5.48% Pb(II); wastewater recovery 70–108%	Very low LODs; integrated device; requires post-LIG Bi deposition and second laser step
This work	PVA-assisted Bi precursor layer stencil-printed on PI before	No chemical PI pretreatment; no ion exchange; no post-LIG Bi drop-casting	CO_2 laser conversion after PVA/Bi printing; RE printing	Complete in-series self-contained sensors	Zn(II), Cd(II), Pb(II)	Linear ranges: 350–10500 nM Zn(II); 300–9000 nM Cd(II)/Pb(II	Calibration slope RSD ≤ 6.0%; artificial sweat recovery 95–112%, RSD ≤ 12%	Broader analyte scope including Zn; complete in-series

laser conversion	and PET-EVA lamination	); LOD: 104 nM Zn(II), 14 nM Cd(II), 37 nM Pb(II)	sensor manufacturing; no KOH pretreatment or post-LIG Bi step
